# Chondrogenesis with Autologous Peripheral Blood Stem Cells for End-Stage Ankle Arthritis: Report of Three Cases

**DOI:** 10.5704/MOJ.2203.022

**Published:** 2022-03

**Authors:** KY Saw, AW Anz, CSY Jee, A Ramlan, A Dawam, SF Low

**Affiliations:** 1 Department of Orthopaedic Surgery, Kuala Lumpur Sports Medicine Centre, Kuala Lumpur, Malaysia; 2 Department of Orthopaedics, Andrews Institute for Orthopaedics and Sports Medicine, Gulf Breeze, United States; 3 Stem Cells R&D, Kuala Lumpur Sports Medicine Centre, Kuala Lumpur, Malaysia; 4 Department of Radiology, Kuala Lumpur Sports Medicine Centre, Kuala Lumpur, Malaysia

**Keywords:** end-stage ankle arthritis, massive chondral defects, chondrogenesis, peripheral blood stem cells, marrow stimulation

## Abstract

End-stage ankle arthritis represents an “unmet medical need”, awaiting an appropriate time for joint arthroplasty or arthrodesis. We report three cases of end-stage ankle arthritis treated along the principles developed for chondrogenesis of the knee joint with autologous peripheral blood stem cells, resulting in reversal of the ankle arthritis. The improvement in clinical outcome measure scores (Ankle Osteoarthritis Scale total score) with a minimum two-year follow-up were comparable to total ankle replacement (TAR), arthroscopic ankle arthrodesis (AAA) and open ankle arthrodesis (OAA).

## Introduction

End-stage ankle arthritis is incapacitating and options are limited to total ankle replacement (TAR), arthroscopic ankle arthrodesis (AAA) and open ankle arthrodesis (OAA). However, these procedures have a high complication rate of 20% to 27%^1^ with 20% requiring further surgery^2^.

We developed the arthroscopic KART (Kuala Lumpur Sports Medicine Centre Articular Regeneration Technology) procedure for chondrogenesis of the knee joint and recently concluded a phase IIB US Food and Drug Administration randomized controlled trial addressing massive knee chondral defects with overwhelming clinical significance^3^. Applying the same principles developed for chondrogenesis with autologous peripheral blood stem cells (PBSC), we report the results of applying KART to address end-stage ankle arthritis.

## Report of Three Cases

**Fig. 1:**
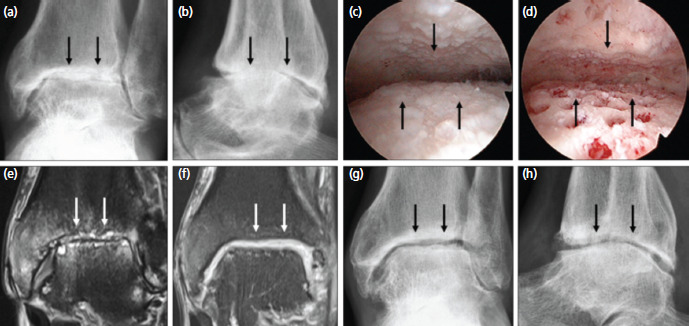
(a) Anteroposterior and (b) lateral radiographs of the left ankle joint showing end-stage arthritis (black arrows). (c) Arthroscopic view of the left ankle joint showing diffuse bone-on-bone cartilage loss (black arrows). (d) View after arthroscopic subchondral drilling of almost the entire ankle joint (black arrows). (e) Coronal MR image showing advanced arthritic changes (white arrows). (f) MR image at two years following chondrogenesis with satisfactory cartilage thickness on both articular surfaces (white arrows). (g, h) Radiographs at two years showing reappearance of the ankle joint space (black arrows). The MR images are fat-suppressed proton density-weighted.

This first case was a 58-year-old man who presented with left ankle pain following a motorcycle accident 30 years previously. He underwent open reduction and internal fixation (ORIF) of the ankle fracture with subsequent removal of the metalwork. Arthroscopic ankle debridement was performed earlier prior to being seen without clinical benefit. Radiographs showed advanced arthritis of the ankle joint (Fig. 1a, 1b).

He underwent left ankle arthroscopy with burring of osteophytes followed by arthroscopic subchondral drilling of almost the entire ankle joint (Fig. 1c, 1d). One week after surgery, PBSC were harvested. The details of the harvesting procedure and cell preparation are outlined in our previous publication^4^. Immediately after the apheresis process, 8ml of fresh PBSC aliquot was mixed with 2ml of HA [Hyalgan; Fidia Farmaceutici, Abano Terme, Italy] and injected into the operated ankle joint under aseptic conditions. Haemarthrosis was aspirated prior to each injection. Subsequently, at 4 weekly intervals, an 8ml aliquot of the thawed cryopreserved PBSC mixed with 2ml HA were injected into the operated ankle joint. Physiotherapy with joint mobilisation commenced one day after surgery, progressing from partial to full weight bearing in 12 months.

At months 6, 12, 18 and 24 following surgery, 3 additional weekly intra-articular injections comprising 4ml of thawed cryopreserved PBSC and 2ml of HA were given. Preoperative MRI image (Fig. 1e) and at two years (Fig. 1f) showed chondrogenesis. This was accompanied by the reappearance of the ankle joint articulation on radiographs (Fig. 1g, 1h).

**Fig. 2:**
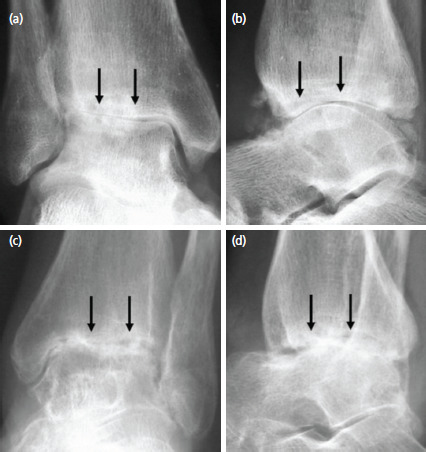
(a) Anteroposterior and (b) lateral radiographs of the right ankle joint of the second case with (c, d) corresponding views of the left ankle joint of the third case. Arrows showing advanced arthritic changes.

The second case was a 43-year-old man who sustained a right ankle fracture and underwent ORIF three years previously with subsequent removal of metalwork (Fig. 2a, 2b).

The third case was a healthy 56-year-old woman who had a soft tissue infection of the left ankle four years previously, resulting in septic arthritis. Arthroscopic and open drainage was performed followed by antibiotics. Six months prior to being seen, she had further ankle arthroscopy with soft tissue and bony biopsies for microbial cultures. There was no evidence of persistent infection and haematological results were normal. The resultant septic arthritis with destruction of the ankle joint is shown (Fig. 2c, 2d).

Both the second and third cases underwent ankle arthroscopic procedures with post-operative regimes similar to the first case. However, the third case did not have the opportunity to receive the month 18 and 24 booster PBSC plus HA. The reason being that she was a medical tourist and was unable to return for further injections due to the lockdown imposed as a result of the COVID-19 pandemic at the time of writing.

**Fig. 3:**
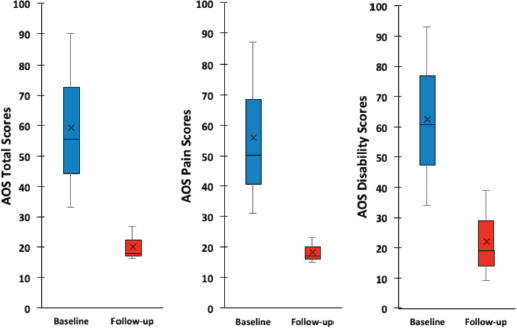
Box plot showing clinical outcome measure scores, preoperatively and at the time of the latest follow-up for the three cases, with a minimum two years follow-up. The top and bottom of the box represent the interquartile range, the line within the box represents the median, the ‘x’ within the box represents the mean and the whiskers indicate the range. AOS scores show greater pain and disability when the number is higher. Improvement of pain and disability is indicated by lower numbers at follow-up.

Fig. 3 and Table I show the pre-operative and the latest follow-up Ankle Osteoarthritis Scale (AOS) scores for the 3 cases. Appreciable improvement of the clinical scores was seen. Statistical analysis was not possible due to insufficient data. There were no reported adverse events with no post-operative infections or deep venous thrombosis.

**Table I:** Patient demographics and improvement in clinical outcome measure scores from baseline to final follow-up in all three cases.

Case	Gender	Age at surgery	BMI at surgery (kg/m^2^)	Period of follow-up (years)	Clinic outcome measure scores improvement		
					AOS total	AOS pain	AOS disability
1	Male	58	24.3	3.3	28.8	35.3	22.1
2	Male	43	25.8	2.8	15.0	14.0	15.0
3	Female	56	18.7	2.2	74.0	64.0	84.0
Mean	-	52	22.9	2.8	39.3	37.8	40.4

## Discussion

It is clear that chondrogenesis with PBSC has a role to play in end-stage ankle arthritis which represents an “unmet medical need” especially in the younger population. Objective evaluation by radiographs and MRI scans charted the healing process and allowed assessment of the entire repair area in a non-invasive manner. The scans revealed no evidence of adverse articular or extra-articular abnormalities. There was no documented infection or major adverse events.

Current options for end-stage ankle arthritis are limited to TAR, AAA and OAA. Ankle arthrodesis results in altered function with the potential of accelerating adjacent joint degeneration. TAR was developed to allow ankle motion, improve gait and preserve adjacent joints. However, these procedures are technically demanding, and have high complications rates. Moreover, salvaging a failed TAR is challenging and the results are worse off when compared to primary arthrodesis^1,2^.

The AOS scores of the reported three cases are presented (Fig. 3 and Table I). This showed improvement of the scores at the follow-up time points as compared to the baseline. The improvement in clinical outcome measure scores (mean difference between the baseline to final follow-up scores) in our three cases are comparable to those reported by Veljkovic *et al*^2^. They reported AOS total scores of 34.4 for TAR group, 38.3 for AAA group and 25.8 for OAA group as compared to ours of 39.32. The AOS pain and disability scores were of the similar range.

Our results are reassuring as these are end-stage cases representing "unmet clinical needs". The main advantage is a one-stage surgical procedure involving arthroscopic subchondral drilling followed by post-operative intra-articular injections of PBSC with HA, without the need for metallic implants. The procedure does not “burn any bridges” for future TAR or ankle arthrodesis if and when the need arises.

Our previous publications have shown the ability of the KART procedure to regenerate resilient hyaline cartilage in massive chondral defects of the knee joint resembling 95% of normal articular cartilage histologically^3-5^. We believe that the same scenario applies to the ankle joint. This concept of osteoarthritis (OA) reversal may not seem plausible but the basic principles and evidence provided to date showed that this is a logical and promising way to move forward^3-5^. Reversal of OA as shown in Fig. 1 and Fig. 2 is within grasp.

Arthroscopic subchondral drilling followed by post-operative intra-articular injections of autologous PBSC plus HA is effective for the management of end-stage ankle arthritis in younger patient cohorts.
